# Salvianolic acid B protects against myocardial ischaemia-reperfusion injury in rats via inhibiting high mobility group box 1 protein expression through the PI3K/Akt signalling pathway

**DOI:** 10.1007/s00210-019-01755-7

**Published:** 2019-12-18

**Authors:** Hanqing Liu, Wei Liu, Huiliang Qiu, Dezhi Zou, Huayang Cai, Qiuxiong Chen, Chaoyang Zheng, Danping Xu

**Affiliations:** 1 Cardiovascular Department, Guangzhou Hospital of integrated Traditional and West Medicine, Guangzhou, 510800 China; 2 Geriatrics Department, Tongde Hospital of Zhejiang Province, Hangzhou, 310012 Zhejiang China; 3 Second Clinical Medical College, Guangzhou University of Chinese Medicine, Guangzhou, 510006 China; 4 Emergency Department, The First Affiliated Hospital, Sun Yat-sen University, Guangzhou, 510080 China; 5 Internal Medicine Department, Guangdong Provincial Hospital of Chinese Medicine, 2nd Affiliated Hospital of Guangzhou University of Chinese Medicine, Guangzhou, 510120 China; 6 Cardiovascular Department, Guangdong Provincial Hospital of Chinese Medicine, 2nd Affiliated Hospital of Guangzhou University of Chinese Medicine, Guangzhou, 510120 China; 7 Guangdong Provincial Academy of Chinese Medical Sciences, Guangzhou, 510006 China

**Keywords:** Sal B, Myocardial ischaemia/reperfusion injury, PI3K/Akt/HMGB1

## Abstract

Salvianolic acid B (Sal B) has a significant protective effect on myocardial ischaemia-reperfusion (I/R) injury. Therefore, the aims of this study were to determine the effects of Sal B on myocardial ischaemic-reperfusion (I/R) injury in rats and to explore whether its underlying mechanism of cardioprotection occurs through activating the expression of the phosphoinositide 3-kinase/protein, kinase B (PI3K/Akt) and inhibiting the expression of high mobility group protein 1 (HMGB1). Ninety Sprague-Dawley rats were randomized into five groups: group 1 (sham-operated), group 2 (myocardial I/R), group 3 (low dose of Sal B+I/R), group 4 (high dose of Sal B+I/R), and group 5 (high dose of Sal B+I/R+LY294002, which is a specific PI3k inhibitor). All I/R rats received 30 min myocardial ischaemia followed by 24-h reperfusion. Cardiac function, infarct size, myocardial injury marker levels, inflammatory response and cardiomyocyte apoptosis as well as Bcl-2, Bax, P-Akt, HMGB1 and TLR4 expression were measured. In the current study, Sal B significantly ameliorated myocardial I/R injury in a dose-dependent manner, ameliorated cardiac function, reduced myocardial infarction size, decreased myocardial injury marker expression, decreased inflammatory responses, reduced apoptosis, activated PI3K/Akt expression and inhibited HMGB1 expression. However, all effects of Sal B were significantly reversed by LY294002. Overall, the present study indicated that Sal B attenuated myocardial I/R injury by activating PI3K/Akt and inhibiting the release of HMGB1 in rats.

## Introduction

Coronary heart disease is a leading cause of death associated with cardiovascular disease. Acute myocardial infarction (AMI)-associated mortality and morbidity are common worldwide (Frank et al. 2012). Early myocardial reperfusion is the preferred treatment for AMI in most clinical settings (Binder et al. 2015). The rapid restoration of blood flow is a critical therapeutic strategy. However, abrupt myocardial reperfusion can sometimes exacerbate tissue injury and is called myocardial ischaemia and reperfusion (I/R) injury (Jennings 2013). The mechanism of myocardial I/R injury is very complex. There are numerous causes of myocardial I/R injury, such as inflammation, apoptosis, mitochondrial dysfunction and oxidative stress (Braunersreuther et al. 2013; Zhu et al. 2015; Li et al. 2016a, b, Wang, Han and Jia 2018)**.**

Danshen, the dried root of *Salvia miltiorrhiza* Bunge, is a Chinese medicinal herb. Salvianolic acid B (Sal B) is an active water-soluble component that can be isolated from *S. miltiorrhiza* Bunge (Chan et al. 2004; Hu et al. 2005; Lam et al. 2007). Sal B exhibited multiple bioactivities, including the reduction of the expression of related inflammatory factors, inhibition of apoptosis and alleviation of oxidative stress (Lv et al. 2015; Zhao et al. 2017). As an abundant bioactive component of *S. miltiorrhiza*, it has been reported that Sal B protects against I/R-induced cerebral injury by reducing cerebral infarct size and improving neurobehavioural functions (Fan et al. 2018). In addition, it has been shown that Sal B has cardiovascular protective effects, due to its potent anti-oxidative capabilities and inhibition of inflammation during cardiovascular injury (Ho and Hong 2011). Additionally, previous studies have demonstrated that Sal B has significant protective effect on myocardial I/R injury by alleviating oxidative stress, reducing calcium overload, improving endothelial function, stabilizing mitochondrial membrane potential and upregulating microRNA-30a (Xue et al. 2014; Deng et al. 2015; Li et al. 2016a, b).

The PI3K/Akt signalling pathway plays an important role in cardioprotection and is involved in regulating cardiomyocyte survival, apoptosis and inflammatory responses (Cao et al. 2013; Yao, Han and Han 2014; Tang et al. 2017). Recently, some studies have reported that endoplasmic reticulum stress-regulated apoptosis and excessive autophagy can be inhibited and the myocardium can be protected from I/R injury through the activating PI3K/Akt signalling pathway (Li et al. 2018; Yu et al. 2019). HMGB1 is a nuclear protein that is passively released from injured cells (Zhou, Li and Mu 2015; Ouyang et al. 2016). It has been confirmed that dexmedetomidine protects cardiomyocytes against hypoxia/reoxygenation-induced necrosis by inhibiting HMGB1-mediated inflammation (Chen et al. 2019).

Previous study has shown that anti-inflammatory effect of B-type natriuretic peptide(BNP) postconditioning during myocardial I/R injury is associated with inhibiting HMGB1 expression through activating of PI3K/Akt signalling pathway (Hu et al. 2014). Furthermore, Celastrol and simvastatin have been reported to protect the myocardium against I/R injury by inhibiting HMGB1 expression via activating PI3K/Akt pathway (Han et al. 2015; Tong et al. 2018).

Therefore, we determined whether Sal B could modulate the release of HMGB1 during myocardial I/R injury and speculated that the cardioprotective mechanism of Sal B against myocardial I/R injury was through the inhibition HMGB1 expression through activating the PI3K/Akt signalling pathway.

## Materials and methods

### Animals

Ninety male Sprague-Dawley rats (250–300 g) were obtained from Guangdong Provincial Medicinal Laboratory Animal Center (SCXK (Yue) 2013-0002, Guangzhou, China). All animals in this study were treated in accordance with the Guide for the Care and Use of Laboratory Animals published by the National Institutes of Health (Bethesda, MD, USA) and the Regulations of Experimental Animal Administrations published by the State Committee of Science and Technology of the People’s Republic of China. The study protocol was approved by the Animal Care Committee of Guangdong Provincial Hospital of Chinese Medicine. All rats used in this study were allowed free access to food and water and were housed in the Animal Center of Guangdong Provincial Hospital of Chinese Medicine.

### Drugs and chemicals

Sal B (HPLC > 98%) was purchased from Chengdu Manst Biotechnology Co. Ltd. 2,3,5-Triphenyltetrazolium chloride (TTC) was purchased from MB-CHEM (USA). Haematoxylin (HE) was purchased from Keygen Biological Technology Co. Ltd., Jiangsu, China. T-Akt (11E7), P-Akt (Ser473) and glyceraldehyde-3-phosphate dehydrogenase (GAPDH) (5174) were purchased from Cell Signaling Technology (USA). The HMGB1 and goat anti-rabbit secondary antibodies were purchased from Abcam (USA). Bcl-2 associated × protein (Bax), Beclin-2 (Bcl-2) and Toll-like receptor 4 (TLR4) were purchased from Proteintech Group, Inc. (USA). LY294002 was purchased from MedChemExpress (China). L-L*actate* dehydrogenase (L-LDH), creatine kinase (CK-MB), tumour necrosis factor-α (TNF-α), interleukin-18 (IL-18), interleukin-1β (IL-1β) and HMGB1 enzyme-linked immunosorbent assay (ELISA) kits were purchased from Wuhan Huamei Bioengineering Co., Ltd. 3,3′-Diaminobenzidine(DAB) was purchased from China Hubei Boster Biotechnology Co., Ltd. Terminal deoxynucleotidyl nick-end labelling (TUNEL) kit and phosphatase inhibitor cocktail were purchased from Roche Group, Inc. (Swiss). RIPA lysis buffer was purchased from Beyotime Institute of Biotechnology (China). BCA protein assay kit was purchased from Thermo (USA). Enhanced chemiluminescence reagents (ECL) was purchased from EMD Millipore (USA).

Sal B was dissolved in 0.9% sodium chloride (NaCl). Different doses of Sal B were administered through intraperitoneal (i.p.) injection immediately after being dissolved in 0.9% NaCl and 30 min before the myocardial ischaemia models were completed.

### Experimental protocols

The rats were randomly assigned to five groups and prepared for different treatments, based on previous studies with modifications (Xue et al. 2014; Qiao and Xu 2016). Group 1 (sham-operated (Sham, *n* = 18)) was not treated, and rats in this group underwent surgical manipulation without ligating left anterior descending coronary artery (LAD). Group 2 (myocardial I/R (I/R, *n* = 18)) rats were ischaemic for 30 min and reperfused for 24 h. Group 3 (low dose of Sal B+I/R (Sal-L, *n* = 18)) rats received 15 mg/kg/day Sal B via intraperitoneal injection for 4 days before operation. Group 4 (high dose of Sal B+I/R (Sal-H, *n* = 18)) rats received 60 mg/kg/day Sal B via intraperitoneal injection for 4 days before operation. Group 5 (high dose of Sal B+I/R+LY294002 (Sal-H+LY, *n* = 18)) rats received 60 mg/kg/day Sal B via intraperitoneal injection for 4 days before operation and LY294002 (an inhibitor of PI3K, 0.3 mg/kg) dissolved in 0.02% dimethyl sulfoxide via caudal vein injection 30 min before LAD ligation. All I/R rats underwent myocardial ischaemia for 30 min followed by reperfusion for 24 h.

### Myocardial I/R model in rats

Rats were anaesthetized with intraperitoneal injection of 10% chloral hydrate (350 mg/kg). After the rats were anaesthetized, they were treated with artificial ventilation with a volume-controlled rodent ventilator (Harvard Apparatus, Model Inspira) at a rate of 60 strokes per minute and monitored with an electrocardiograph. Briefly, left thoracotomy and pericardiotomy were performed, and 7–0 nylon slip-knot sutures were inserted around the LAD. Then, myocardial ischaemia was induced. The myocardial ischaemia rats were monitored, and their condition was confirmed by ST segment elevation in an electrocardiogram (ECG). Then, an I/R injury animal model was induced by ligating the LAD for 30 min followed by 24-h reperfusion (Samsamshariat, Samsamshariat and Movahed 2005).

### Cardiac function assessment

After the rats were anaesthetized, cardiac function was assessed by cardiac output (CO), left ventricular ejection fraction (LVEF), fractional shortening (FS), stroke volume (SV) and heart rate (HR). These parameters were monitored and digitally processed throughout an echocardiograph using a Visual Sonics Vevo 2100 system (Canada).

#### Infarct size assessment

Myocardial infarct size was measured with a 1% solution of TTC stain. In brief, after 24 h of reperfusion, the heart was removed and frozen at − 20 °C. Then, each heart was horizontally sliced into 5-mm slices. These slices were incubated in 1% TTC in phosphate buffer for 15–20 min at 37 °C photographed used a digital camera (Canon, Japan). After TTC staining, the white part represented myocardial infarction area, and the red part represented no infarction. Myocardial infarct size was analysed using ImageJ 1.36 software from the National Institutes of Health. Infarct size was expressed as the following percentage: infarction area/total left ventricular area (INF/LV). The total left ventricular area corresponds to the risk area because global ischaemia was induced (Wu et al. 2014).

#### ELISA analysis

After 24 h of reperfusion, blood samples were collected from the abdominal aorta and centrifuged at 4 °C for 15 min at a speed of 1000*g*. The supernatant was stored at − 80 °C until ELISA analysis was performed. The levels of L-LDH, CK-MB, TNF-α, IL-18, IL-1β and HMGB1 were measured by using commercial ELISA kits following the manufacturer’s instructions.

### Immunohistochemical staining

The myocardial tissue specimens of rats were fixed in a 4% paraformaldehyde solution, dehydrated and embedded in paraffin. The tissue samples were cut into 3-μm-thick sections. These sections were dewaxed with gradient xylenol solutions and repaired with a sodium citrate antigen solution. Endogenous catalase activity was inhibited with 3% hydrogen peroxide, and the sections were sealed with 5% Bovine Serum Albumin (BSA) at 37 °C for 1 h. Then, the samples were incubated overnight with HMGB1 and TLR4 primary antibodies at 4 °C. After rinsing with PBS three times (3 min each time), the samples were treated with a biotin goat anti-rabbit secondary antibody solution, incubated at 37 °C for 30 min and washed with PBS 3 times (3 min each time). In addition, the samples were treated with a DAB dye solution for 1 to 2 min and washed with PBS once. The samples were stained again with HE for 1~2 min, washed with water, dehydrated with gradient ethanol xylene solutions and sealed with neutral balata. Finally, the distribution of immunopositive cells was evaluated under a light microscope. All the analyses were performed with Image-Pro Plus 6.0 software.

### Apoptosis analysis

Myocardial apoptosis was analysed by using a TUNEL kit following the manufacturer’s protocol. In brief, after 24 h of reperfusion, the heart was excised. The anterior wall of the left ventricle of the heart was removed, fixed with 4% formaldehyde, embedded in paraffin, cut into 5-μm sections and stained with TUNEL. The apoptotic cardiomyocytes of rats were visualized under an optical microscope, and the apoptotic cells were counted using the Image-Pro Plus 6.0 software from the National Institutes of Health. The percentages of TUNEL-positive cells were calculated by dividing the total number of apoptotic cells by the total number of cells in each field.

### Western blotting

The left ventricle tissues of rats were homogenized in 1 ml RIPA lysis buffer containing 1 mM phenylmethanesulfonyl fluoride and 1% phosphatase inhibitor cocktail and then centrifuged at 14000 rpm at 4 °C for 15 min. The supernatants were collected and used for western blot assays. Protein concentrations were quantified by a BCA protein assay kit. Proteins (15–20 μg) were separated by 10–12% sodium dodecyl sulfate polyacrylamide gel electrophoresis (SDS-PAGE) and then transferred onto polyvinylidene fluoride membranes (EMD Millipore, USA). Following blocking for 2 h with 5% skim milk at room temperature, the membranes were incubated with primary antibodies overnight at 4 °C. After washing three times with TBST, the membranes were incubated with secondary antibodies at room temperature for 1.5 h. The protein bands were detected by ECL and the results were quantified by Image Lab 5.2.1 software (Bio-Rad, Hercules, USA). The primary antibodies used included T-Akt (11E7), P-Akt (Ser473), GAPDH (5174), HMGB1, Bcl-2 and Bax.

### Statistical analysis

All the data are expressed as mean ± SD. SPSS 17.0 software was used for statistical analysis. Among the statistical analyses, the normal distribution and homogeneity of variance measurements were measured by one-way ANOVA, and the test level was alpha = 0.05. At *P* < 0.05, the difference was considered statistically significant. The Kruskal-Wallis test was used for data with non-homogeneous variance. *T* test or rank sum test was used for comparisons between two groups.

## Result

### Sal B ameliorated cardiac function

**Fig. 1:**
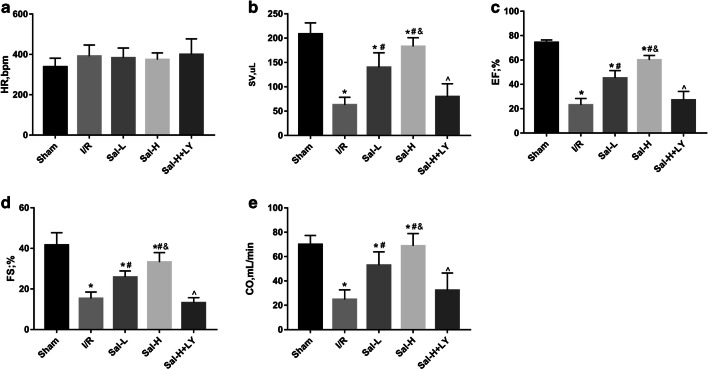
Cardiac function. Sal B ameliorated cardiac function, but this effect was abolished by LY294002 treatment. The values of HR (**a**), SV (**b**), LVEF (**c**), FS (**d**) and CO (**e**) were monitored and digitally processed throughout echocardiography digitally processed throughout an echocardiography. Heart rhythm (HR), cardiac output (CO), ejection fraction (EF), fractional shortening (FS), stroke volume (SV). Sham-operated rats (Sham, *n* = 18), myocardial ischaemia reperfusion injury rats (I/R, *n* = 18), I/R rats treated with low dose of Sal B (Sal-L, *n* = 18), I/R rats treated with high dose of Sal B (Sal-H, *n* = 18), Sal-H rats treated with the PI3K inhibitor LY294002 before LAD ligation (Sal-H+LY, *n* = 18). All data are expressed as mean ± SD, *P < 0.05 versus Sham, ^#^*P* < 0.05 versus I/R, ^&^*P* < 0.05 versus Sal-L, ^^^*P* < 0.05 versus Sal-H

The values of HR, SV, LVEF, FS and CO in each group are shown in Fig. 1. There were no significant differences among the HR values of the groups. The SV, LVEF, FS and CO values of the I/R group were significantly decreased compared with those of the sham group (*P* < 0.05). After treatment with Sal B in the I/R group, the SV, LVEF, FS and CO values significantly increased (*P* < 0.05). Compared with Sal B-L group, the SV, LVEF, FS and CO values of Sal B-H group significantly increased (*P* < 0.05). However, after adding LY294002 to the Sal-H group, the SV, LVEF, FS and CO values were significantly decreased (*P* < 0.05).

### Sal B reduced myocardial infarction size

**Fig. 2:**
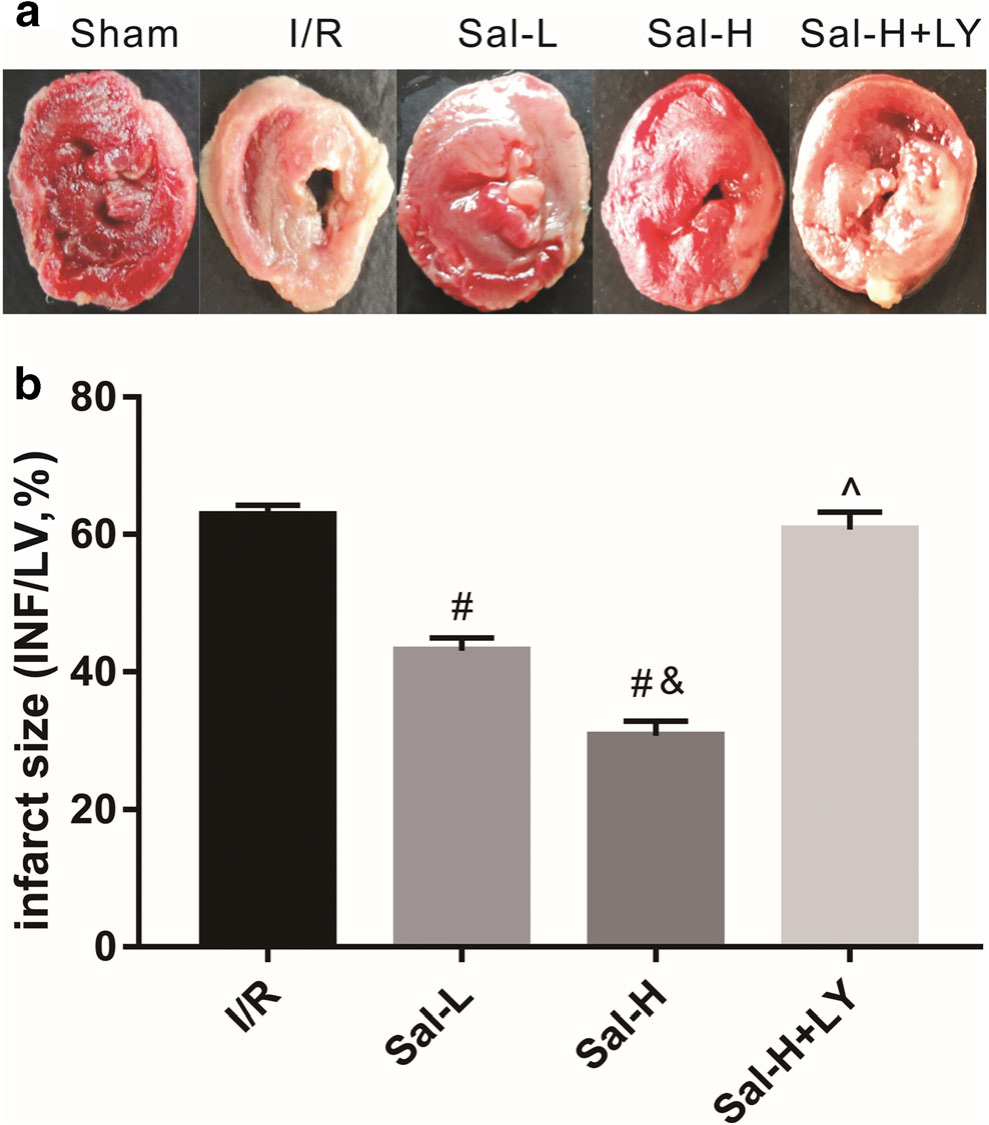
Myocardial infarct size. Sal B reduced the size of myocardial infarction, but this effect was reversed by LY294002 treatment. Myocardial infarct size was measured with TTC staining, the white part represented myocardial infarction (**a**) and the myocardial infarct size was analysed (**b**). All data are expressed as mean ± SD, ^#^*P* < 0.05 versus I/R, ^&^*P* < 0.05 versus Sal-L, ^^^*P* < 0.05 versus Sal-H. INF, infarction area. LV, total left ventricular area

As shown in Fig. 2, no myocardial infarction was found in the sham group. Compared with the sham group, rats in the I/R group had clear infarction. Compared with the I/R group, the percentage of myocardial infarction area in the Sal-L and Sal-H groups was significantly decreased (*P* < 0.05). Meanwhile, the percentage of infarction area was significantly decreased in Sal-H group compared with the Sal-L group (*P* < 0.05). These results suggested that Sal B reduced infarction area in a dose-dependent manner. However, the reduction of the percentage of infarction area was abolished by LY294002 treatment in the Sal-H group (*P* < 0.05), and the infarction area following LY294002 treatment was similar to that of I/R group.

### Sal B reduced myocardial injury and inflammatory response

**Fig. 3:**
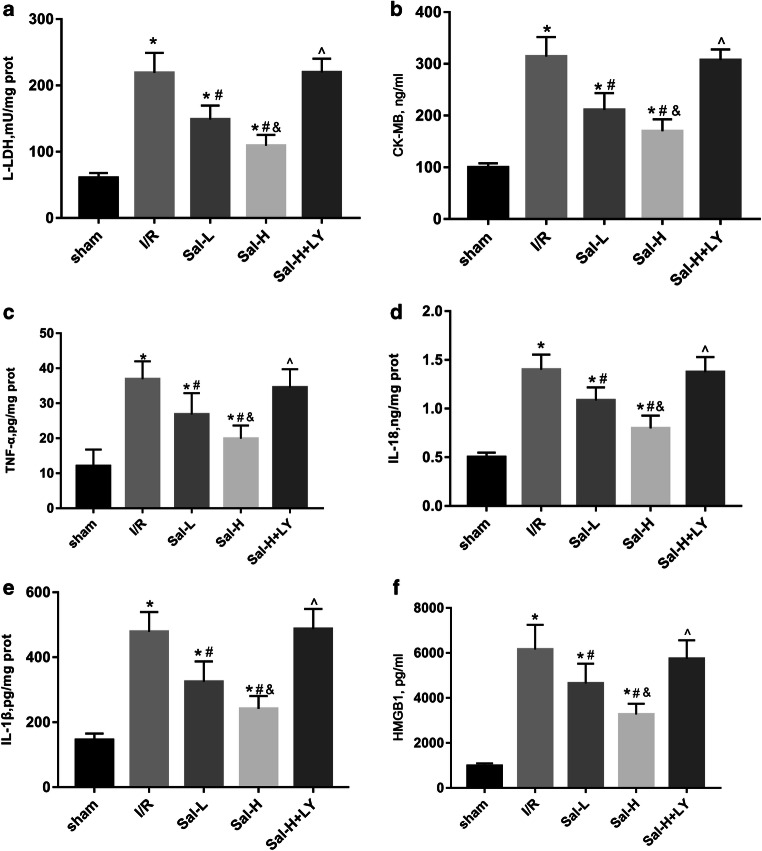
Myocardial injury markers and inflammatory response. Sal B alleviated the level of myocardial injury markers and inflammatory response, while LY294002 has a partial reversal effect. The levels of L-LDH (**a**) and CK-MB (**b**) and the expression of TNF-α (**c**), IL-18 (**d**), IL-1β (**e**) and HMGB1 (**f**) were measured by using enzyme-linked immunosorbent assay (ELISA). All data are expressed as mean ± SD, *P < 0.05 versus Sham, ^#^*P* < 0.05 versus I/R, ^&^*P* < 0.05 versus Sal-L, ^^^*P* < 0.05 versus Sal-H

L-LDH, CK-MB, TNF-α, IL-18, IL-1β and HMGB1 levels were measured to determine whether Sal B attenuated myocardial I/R injury after 24 h of reperfusion (Fig. 3). Compared with the sham group, the levels of L-LDH, CK-MB, TNF-α, IL-18, IL-1β and HMGB1 in the I/R group were markedly elevated (*P* < 0.05). Compared with the I/R group, treatment of the I/R group with Sal B (15 and 60 mg/kg) inhibited the increased L-LDH, CK-MB, TNF-α, IL-18, IL-1β and HMGB1 levels in a dose-dependent manner (*P* < 0.05). However, the levels of L-LDH, CK-MB, TNF-α, IL-18, IL-1β and HMGB1 in the I/R group treated with Sal B (60 mg/kg) were significantly abolished by LY294002 treatment (*P* < 0.05). These results demonstrated that Sal B had a protective effect against myocardial I/R injury and inflammatory response, while LY294002 had a partial reversal effect on myocardial I/R injury (*P* < 0.05).

**Fig. 4:**
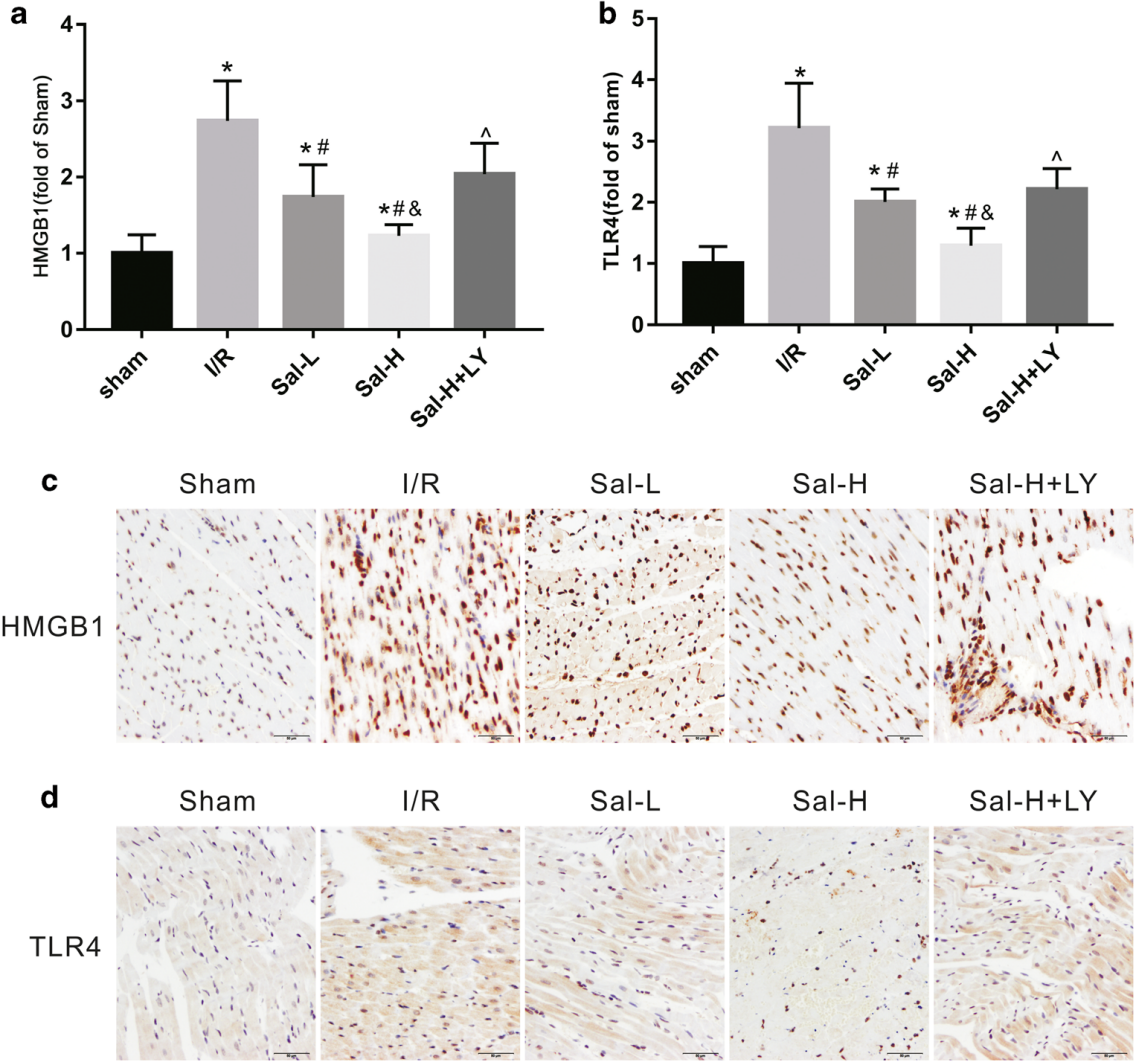
Immunohistochemical detection of HMGB1 and TLR4 expression. Sal B attenuated the expression levels of HMGB1 (**a**) and TLR4 (**b**) in rats with myocardial I/R injury, but LY294002 abolished this effect. Immunopositive cells distribution of HMGB1 (**c**) and TLR4 (**d**) were evaluated under light microscope. All data are expressed as mean ± SD, *P < 0.05 versus Sham, ^#^*P* < 0.05 versus I/R, ^&^*P* < 0.05 versus Sal-L, ^^^*P* < 0.05 versus Sal-H

An immunohistochemical method was used to detect the expression of HMGB1 and TLR4 in each group (Fig. 4). The positive staining for HMGB1 and TLR4 was lower in rats with myocardial I/R injury than in rats without I/R injury. Under light microscopy, myocardial cells with varying amounts of brown granules were HMGB1- and TLR4-positive cells while cells without this staining pattern were negative for HMGB1 and TLR4. Compared with the sham group, the number of HMGB1- and TLR4-positive cells were significantly increased in the I/R group (*P* < 0.05), while those in the Sal B group were significantly decreased in a dose-dependent manner compared to the I/R group (*P* < 0.05). However, the effect of Sal B was reversed by treatment with LY294002 (*P* < 0.05).

### Sal B suppressed myocardial apoptosis

**Fig. 5:**
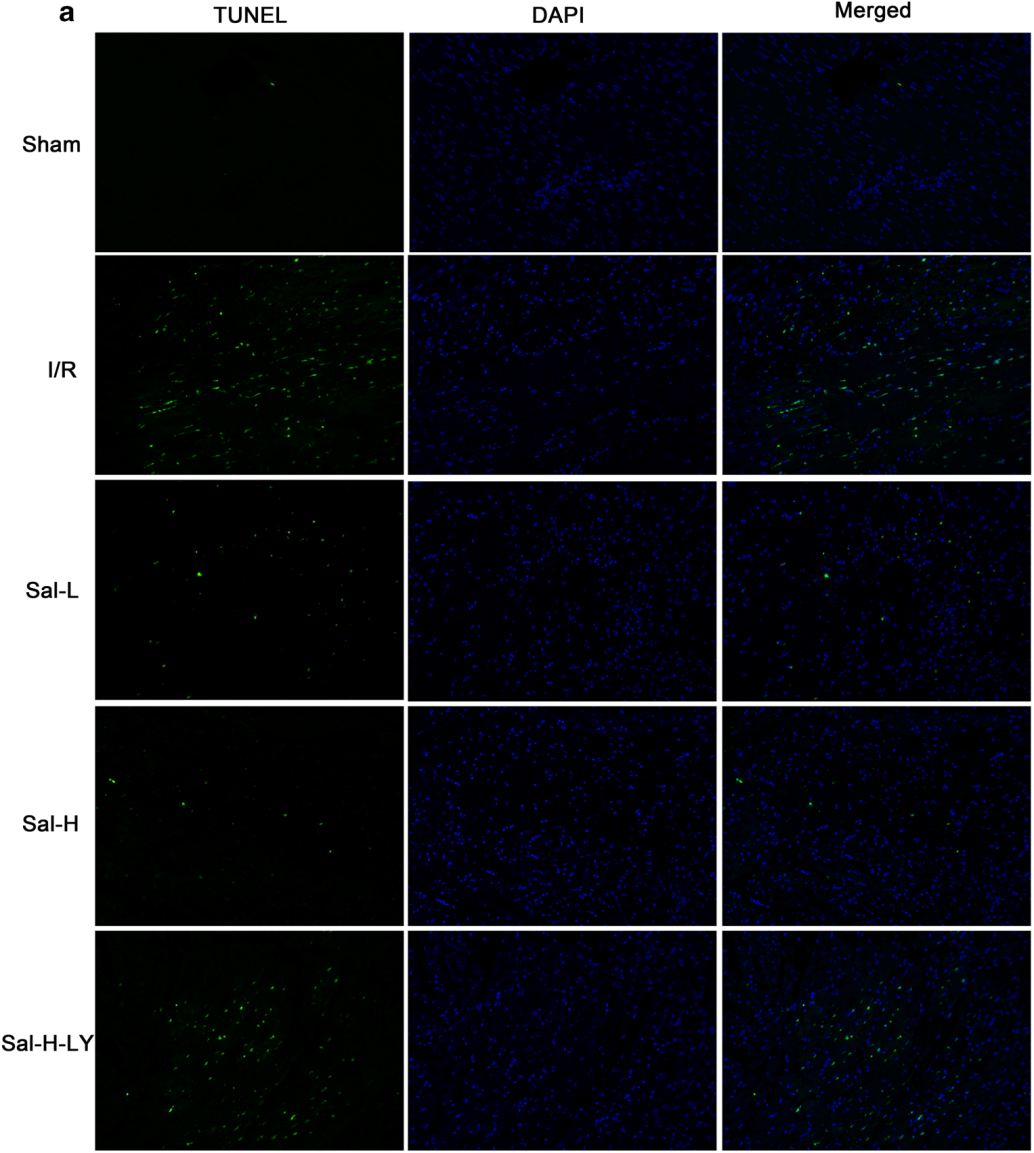

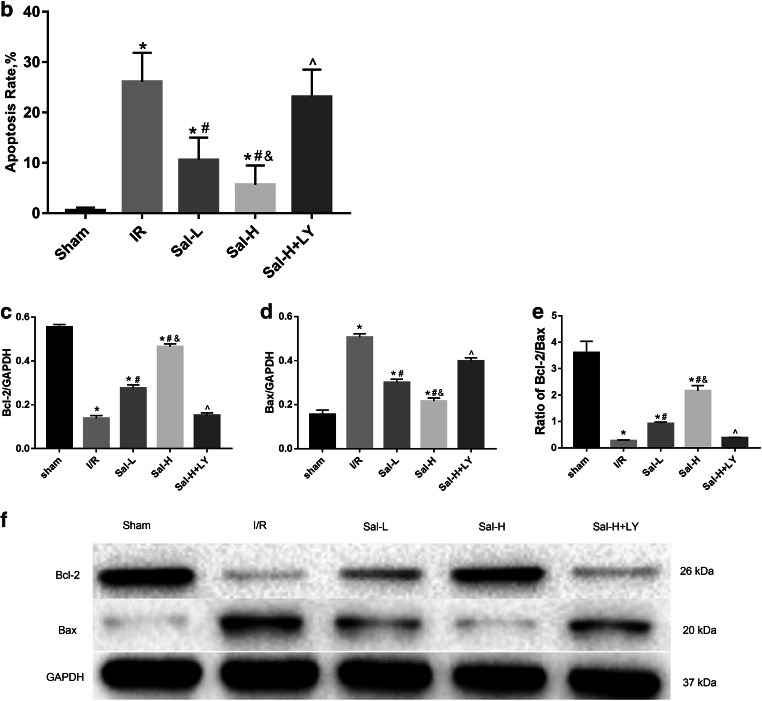
Myocardial apoptosis. Sal B suppressed myocardial apoptosis (**a**) in the I/R group. However, this anti-apoptotic effect was reversed by the administration of LY294002. The rate of apoptotic cells (**b**) was analysed with TUNEL kit and the expression levels of Bcl-2 protein (**c** and **f**) and Bax protein (**d** and **f**) and the ratio of Bcl-2/Bax (**e**) were measured with western blotting. All data are expressed as mean ± SD, *P < 0.05 versus Sham, ^#^*P* < 0.05 versus I/R, ^&^*P* < 0.05 versus Sal-L, ^^^*P* < 0.05 versus Sal-H

The percentage of cardiomyocyte apoptosis in all experimental groups was assessed by TUNEL staining (Fig. 5a, b). Compared with the sham group, the number of TUNEL-positive cells was significantly increased in the I/R group and remarkably reduced in the Sal B treatment group (*P* < 0.05). Moreover, the anti-apoptotic effect of Sal B also occurred in a dose-dependent manner (*P* < 0.05). However, this anti-apoptotic effect was significantly reversed by the administration of LY294002 (*P* < 0.05). Next, we examined the expression of the apoptosis-related proteins Bcl-2 and Bax to further elucidate the underlying mechanism of the anti-apoptotic effect of Sal B (Fig. 5c, d). Compared with the sham group, the Bcl-2 expression level and the Bcl-2/bax ratio of the I/R group were significantly decreased (*P* < 0.05), while the Bax expression level was remarkably increased (*P* < 0.05). However, after treatment with Sal B, the expression of Bcl-2 and the ratio of Bcl-2/Bax significantly increased and the expression of Bax decreased (*P* < 0.05). These results showed that Sal B could effectively reduce cardiomyocyte apoptosis. In contrast, the anti-apoptotic effect of Sal B was partly reversed by LY294002 treatment (*P* < 0.05).

### Sal B activated the PI3K/Akt pathway and suppressed HMGB1 expression

**Fig. 6:**
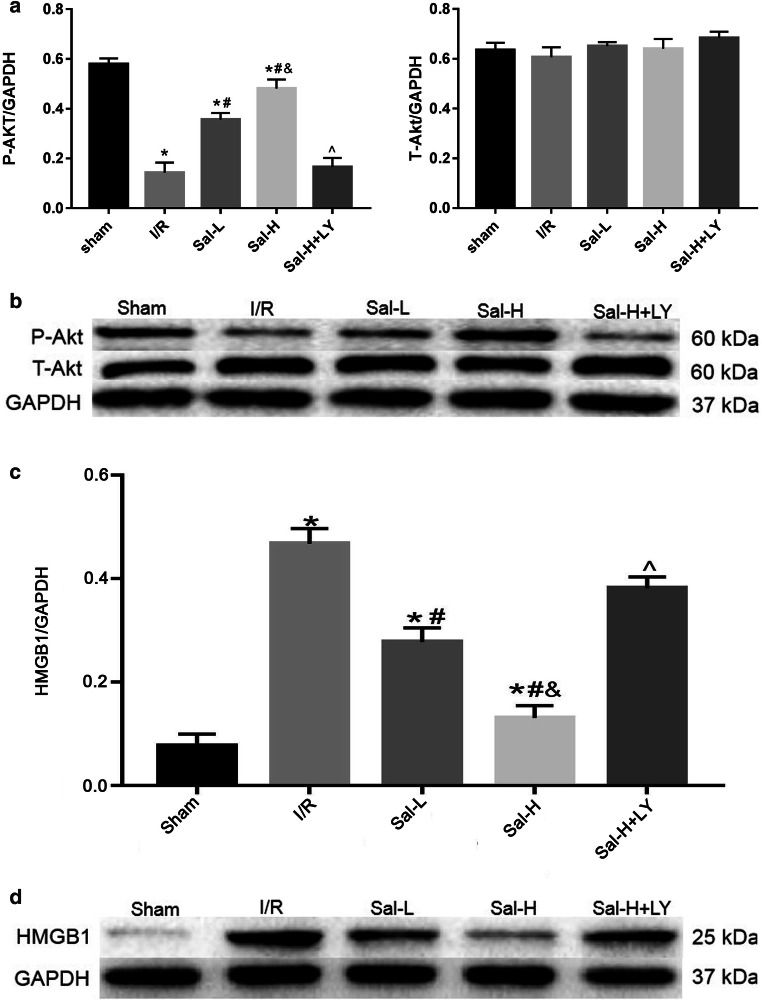
PI3K/Akt/HMGB1 signalling pathway. Sal B activated the PI3K/Akt pathway and suppressed HMGB1 expression. But the effects were blocked by treatment with LY294002. Expression of T-Akt(11E7) (**a** and **b**), P-Akt(Ser473) (**a** and **b**) and HMGB1 (**c** and **d**) protein was measured with western blotting. All data are expressed as mean ± SD, *P < 0.05 versus Sham, ^#^*P* < 0.05 versus I/R, ^&^*P* < 0.05 versus Sal-L, ^^^*P* < 0.05 versus Sal-H

To further investigate the molecular mechanism underlying Sal B-mediated cardioprotection, we determined the expression of P-Akt and HMGB1 protein by western blotting (Fig. 6). Compared with the sham group, the P-Akt protein level was markedly decreased (Fig. 6a, b) and the HMGB1 protein level was significantly increased in the I/R group (Fig. 6c, d) (*P* < 0.05). However, with Sal B treatment, the decrease in the P-Akt protein level and the increase in the HMGB1 protein level were both inhibited (*P* < 0.05). These results suggest that Sal B plays a cardioprotective role in myocardial I/R injury. Moreover, Sal B affected the expression of these proteins in a dose-dependent manner (*P* < 0.05). However, all the effects of Sal B were significantly blocked by treatment with LY294002 (*P* < 0.05). In addition, there was no difference in the T-Akt expression of each group.

## Discussion

*S. miltiorrhiza*, as a traditional Chinese herb, has been widely used for the treatment of ischaemic heart disease in China (Ho and Hong, 2011), and its therapeutic effect mainly relies on its chemical components. Sal B, one of the active components extracted from *S. miltiorrhiza*, has been demonstrated to significantly alleviate myocardial I/R injury in a dose-dependent manner (Ji, Tan and Zhu 2000; Xue et al. 2014; Deng et al. 2015).

In this study, Sal B can significantly ameliorate cardiac dysfunction, reduce the release of myocardium enzyme and decrease infarction size, suggesting that Sal B could produce a protective effect in I/R rats. This may be related to the effects of Sal B, which can attenuate lung and brain injury in rats through inhibiting apoptosis and inflammation (Lv et al. 2015; Zhao et al. 2017). In our findings, it was demonstrated that Sal B treatment can remarkably reduce the infarction size and increase the blood supply of the coronary artery. These results suggested that Sal B could improve cardiac function and reduce the infarction size in I/R model rats. Therefore, further investigation will be necessary to dissect the mechanisms for the potential beneficial clinical applications of Sal B.

Myocardial I/R injury is a complex pathological process, involving various potential mechanisms. It has been proven that myocardial inflammation and apoptosis are the main factors in myocardial I/R injury (Qiao and Xu 2016; Zhang, Liu and Geng 2018). It was also reported that inflammatory cytokines can damage myocardium and may even lead to a systemic inflammatory response (Trachtenberg and Hare 2017).

**Fig. 7:**
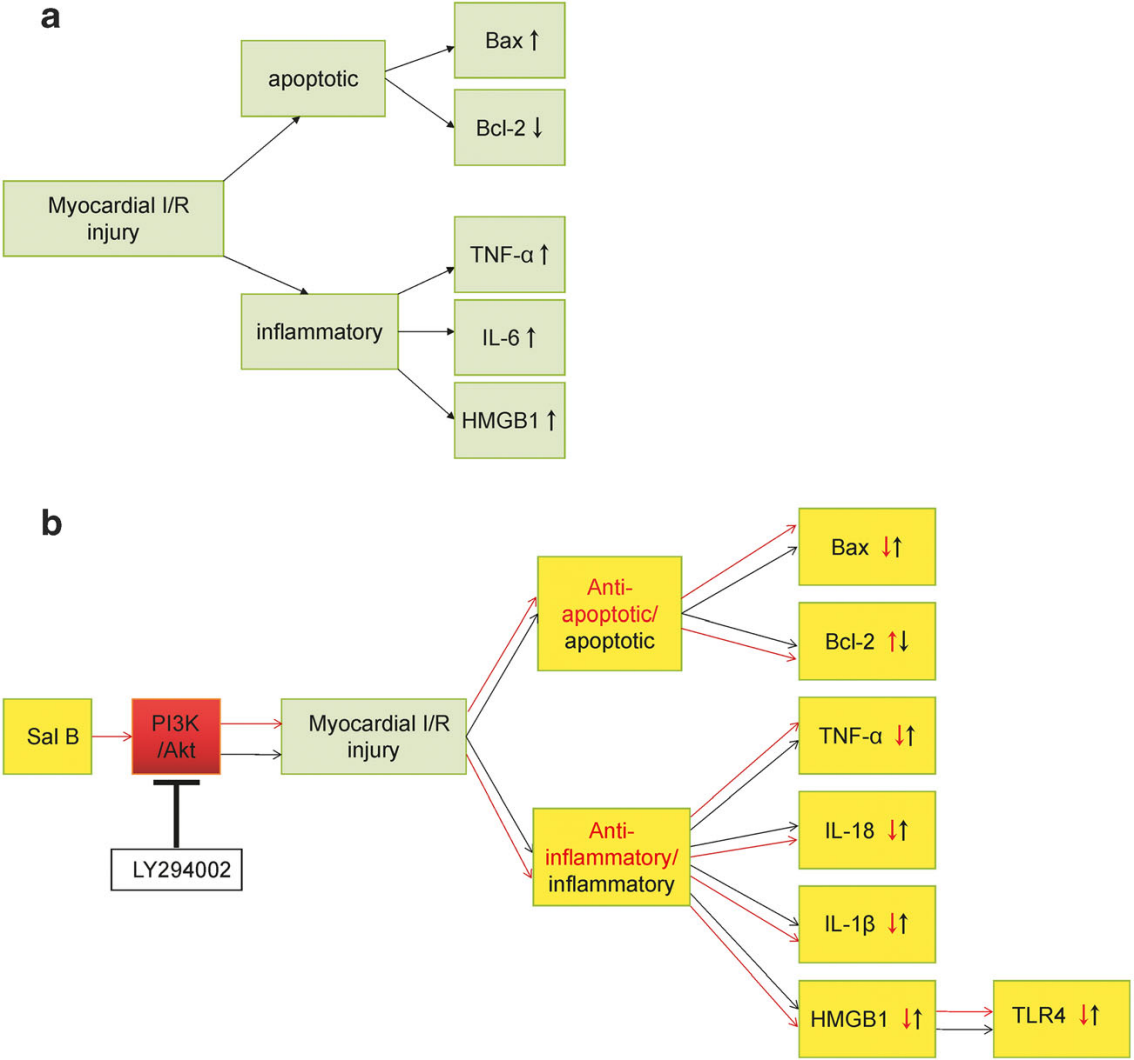
The hypothesis that Sal B protects myocardium through inhibiting the expression of HMGB1 via activating PI3k/Akt signalling pathway in myocardial I/R injury. Previous studies reported that myocardial I/R injury can induce the release of inflammatory cytokines such as TNF-α, IL-6 and HMGB1, and induce cardiomyocyte apoptosis, resulting in the decrease of Bcl-2 and the increase of Bax (**a**). In our study, Sal B alleviates myocardial inflammation by reducing the elevation of these inflammatory cytokines (TNF-α, IL-18, IL-1β, HMGB1 and TLR4) during myocardial I/R injury. Additionally, Sal B can inhibit cardiomyocyte apoptosis by inhibiting the expression of Bcl and Bax. All effects of Sal B are reversed by LY294002 (**b**). In panel b, the red arrow represents the anti-inflammatory and anti-apoptotic effects of Sal B through activating PI3K/Akt pathway on myocardial I/R injury. The black arrow represents that the effect of Sal B is reversed after LY294002. Sal B, Salvianolic acid B. PI3k/Akt, phosphoinositide 3-kinase/protein, kinase B. HMGB1, high mobility group protein 1. TNF-α, tumour necrosis factor-α. IL-18, interleukin-18. IL-1β, interleukin-1β. TLR4, toll-like receptor 4. Bcl-2, beclin-2. Bax, bcl-2 associated × protein. LY294002, an inhibitor of PI3K

During myocardial I/R injury, various pro-inflammatory factors are released, which leads to the release of inflammatory mediators such as interleukin (IL) and TNF-α, which can also release more inflammatory cytokines, cause inflammatory response and aggravate myocardial injury (Kalogeris et al. 2016). Previous studies have shown that myocardial I/R injury can induce the release of inflammatory cytokines such as TNF-α, IL-6 and HMGB1 (Wang et al. 2013; Hu et al. 2014) (Fig. 7a). We examined the expression of TNF-α, IL-18, IL-1β and HMGB1 (Fig. 7b) and found that Sal B could inhibit the level of these myocardial inflammatory cytokines. This result demonstrated that Sal B may protect against myocardial injury by reducing the elevation of these inflammatory cytokines.

In addition, cardiomyocyte apoptosis is considered to be a key factor leading to myocardial I/R injury. Some studies have shown that the role of the Bcl-2 protein family is crucial in the process of cell apoptosis gene regulation (Xie et al. 2001) (Fig. 7a). We detected the expression of pro-apoptotic (Bax) and anti-apoptotic factors (Bcl-2) as well as the apoptosis rate in each experimental group. In our study, the expression of Bcl-2 significantly decreased, while the Bax expression remarkably increased in the I/R group (Fig. 7b). The results showed that Bcl-2 and Bax play different roles in myocardial I/R injury. The experimental results also showed that the expression of Bax decreased and the expression of Bcl-2 increased after treatment with Sal B, indicating that Sal B can mediate the expression of apoptosis-related genes and exert an anti-apoptotic role in cardiomyocytes. However, the anti-apoptotic effect of Sal B was partly reversed by LY294002 treatment, suggesting that the regulation of apoptotic expression was related to the PI3K/Akt signalling pathway (Fig. 7b).

HMGB1, as a nuclear protein released by necrotic or apoptotic cells, is a novel pro-inflammatory cytokine (Scaffidi, Misteli and Bianchi 2002; Bell et al. 2006). Under pathological conditions, HMGB1 can be actively or passively secreted outside of the cell, promoting inflammatory response (Scaffidi et al. 2002; Yamada and Maruyama 2007). Some recent research has shown that HMGB1 plays an important role in myocardial I/R injury by regulating inflammatory factors (Andrassy et al. 2008; Hu et al. 2010; Wang et al. 2012). More recent studies have reported that the inhibition of HMGB1 expression by minocycline and dobutamine alleviates inflammatory responses and myocardium apoptosis in myocardial I/R injury (Wang et al. 2013; Cheng et al. 2018).

TLR4 (receptor of HMGB1), as an inflammatory factor, has been shown to play a detrimental role in myocardial I/R injury (Wang et al. 2016; Yang et al. 2018). Studies have also shown that the activation of TLR4 can aggravate myocardial inflammation and the effective inhibition of TLR4 may be the key to preventing adverse myocardial remodelling (Vilahur and Badimon 2014; Lee, Hutchinson and Saint 2016). In our present study, we found that the expression of HMGB1 and TLR4 was significantly increased in the I/R group but sharply was decreased with Sal B treatment. Based on the above studies and our current findings, we speculated that HMGB1 and TLR4 may promote the inflammatory response in myocardial I/R injury and that Sal B could inhibit myocardial I/R injury through the inhibition of HMGB1 and TLR4 expression.

PI3K/Akt is a well-known signalling pathway involved in myocardial protection (Hausenloy and Yellon 2004). Several studies have shown that the activation of the PI3K/Akt signalling pathway attenuated myocardial I/R injury by inhibiting the inflammatory response, endoplasmic reticulum stress-regulated apoptosis and autophagy (Xu et al. 2018; Ye et al. 2018; Yu et al. 2019). However, it was unclear whether the PI3K/Akt pathway mediates the cardioprotective effects of Sal B.

In the present study, we found that after adding LY294002 treatment to the group treated with a high dose of Sal B, the size of myocardial infarction, the levels of myocardial necrosis and myocardial inflammation markers and the expression of HMGB1 were significantly increased. These results indicated that the cardioprotective effect of Sal B was reversed by LY294002 and that the cardioprotective mechanism of Sal B was involved in the activation of the PI3K/Akt signalling pathway and the inhibition of HMGB1 expression.

It is reported that rats and humans have highly similar genomes, with a similarity of 90%. Its heart structure is also highly similar to that of humans (Gibbs et al. 2004). Previous reports have indicated that inflammation and apoptosis contribute to the initiation and development of I/R injury (Nakano et al. 2016, Schubert et al. 2016). It is confirmed that the most robust end point in experimental animal studies on cardioprotection is infarct size (Botker et al. 2018; Lindsey et al. 2018) and cardioprotective effects beyond the immediate reduction of infarct size are thus not considered, that is, on delayed cell death (by apoptosis or autophagy rather than necrosis), inflammation, repair, remodelling, all of which are, however, essential for clinical outcome studies (Jones et al. 2015). Our result proved Sal B reduced myocardial infarction size by means of reducing myocardial injury and inflammatory response and suppressing myocardial apoptosis.

To validate Sal B could improve cardiac function and reduce the infarction size in I/R model rats, further studies focused on pathological mechanisms and therapeutic reactions are worthy of investigation. We must admit that in the field of cardioprotection, substantial gaps remain between experimental studies aiming at the identification of novel mechanisms and studies providing robust preclinical data which is worth to be tested in humans. Despite the advances represented here, we are clearly still at the beginning of the translation from numerous successful animal experiments to clinical practice on cardioprotection. Much of the additional data needed to complete this story will come from other species, distantly related to rat. Future trials must focus on phase II dose and time data, and recruit patients who have truly a chance to benefit from cardioprotection.

## Study limitations

Our results suggested that Sal B can attenuate myocardial I/R injury by reducing myocardial inflammation and apoptosis. And the cardioprotective mechanism of Sal B on myocardial I/R injury was through reducing the release of HMGB1 through activating the PI3K/Akt signalling pathway. Inevitably, the present study has shortcomings. Firstly, in the present study, quantification of inflammatory mediators was investigated at only one time point (24 h after reperfusion). Indeed, it is not clear whether myocardial I/R injury causes the response of myocardial inflammatory mediators or whether the release of inflammatory mediators leads to myocardial I/R injury. Further research will be needed to determine the expression of these inflammatory factors in a time-dependent manner. Secondly, when assessing myocardial infarct size, the experimental criterion method is the blue dye coloration for delineating infarct area at risk and the identification of viable myocardium by TTC. However, we did not determine the area at risk using blue staining in our experiment. Thirdly, our study provides a potential mechanism for myocardial protection in myocardial I/R injury. However, in the absence of any intervention, different animals, animals of different ages, diets, sources, different laboratory operations and the outcome will be affected. The infarct size of mice, rabbits and pigs can be significantly reduced after ischemic preconditioning, but the release time of troponin is different (Jones et al. 2015). There are significant differences among different species. It is not enough for us to study only one animal model of I/R. Fourthly, it is undeniable that experimental studies in rats can provide reliable preclinical data and deserve to be tested in humans. But this program is to consequence of the high cost of converting rat data to humans/patient data, and the pressing need to further improved and enhanced, such as adding the risk factors, considering the lack of adequate phase II dose and time studies and expanding the number of experimental subjects and scope. However, with the advent of low-cost rat experiments, it is possible to apply for research funding to expand the experiment to bring it closer to current human data.

## Conclusions

The present study indicated that Sal B preconditioning attenuated myocardial I/R injury by reducing myocardial inflammation and apoptosis. In addition, our findings suggest that Sal B attenuated myocardial I/R injury by activating PI3K/Akt signalling pathway and inhibiting the release of HMGB1 in rats.
